# The Relationship Between the Ganglion Cell–Inner Plexiform Layer, Retinal Nerve Fiber Layer, and Photopic Negative Response in Newly Diagnosed Pituitary Macroadenoma: 12-Month Prospective Follow-Up Study

**DOI:** 10.3390/cancers17091542

**Published:** 2025-05-01

**Authors:** Monika Sarnat-Kucharczyk, Dorota Pojda-Wilczek, Ewa Mrukwa-Kominek, Beata Kos-Kudła, Małgorzata A. Janik, Paweł Janik

**Affiliations:** 1 Department of Ophthalmology, Faculty of Medical Sciences in Katowice, Medical University of Silesia, 40-752 Katowice, Poland; pojda-wilczek@wp.pl (D.P.-W.); emrowka@poczta.onet.pl (E.M.-K.); 2 Kornel Gibinski University Clinical Centre, 40-514 Katowice, Poland; beatakos@ka.onet.pl; 3 Department of Endocrinology and Neuroendocrine Tumors, Medical University of Silesia, 40-514 Katowice, Poland; 4 Institute of Biomedical Engineering, Faculty of Science and Technology, University of Silesia in Katowice, 41-205 Sosnowiec, Poland; malgorzata.janik@us.edu.pl (M.A.J.); pawel.janik@us.edu.pl (P.J.)

**Keywords:** optical coherence tomography, ganglion cell–inner plexiform layer, electroretinography, photopic negative response, W-ratio

## Abstract

Background/Objectives: This prospective study evaluates the relationship between the ganglion cell–inner plexiform layer (GCIPL), retinal nerve fiber layer (RNFL), and photopic negative response (PhNR) in patients with newly diagnosed pituitary macroadenomas over 12 months. Methods: A total of 40 patients (80 eyes) were included, divided into a treatment group of 27 patients (54 eyes), receiving pharmacological and/or surgical intervention, and an observation group of 13 patients (26 eyes), with non-functional pituitary adenomas (NFPAs) that did not require treatment. Results: Key findings indicate a significant improvement in best corrected visual acuity (BCVA) after 12 months in the treatment group (*p* = 0.02) and a significant reduction in RNFL thickness in multiple quadrants (*p* < 0.01). Moreover, PhNR amplitude and W-ratio significantly increased in the treatment group (*p* < 0.0001). In the observation group, only GCIPL Inferior (*p* = 0.0470) and PhNR W-ratio (*p* = 0.0015) showed significant differences. Between-group comparisons showed significant differences in RNFL Nasal quadrant at baseline (*p* = 0.0017) and after 12 months (*p* = 0.0150). PhNR amplitude and W-ratio also differed significantly between groups at 12 months (*p* = 0.0012 and *p* = 0.0016, respectively). Correlations between OCT and ERG parameters were weak at baseline and diminished over time. Conclusions: These findings suggest that GCIPL, RNFL, and PhNR analyses may be useful for monitoring disease progression and guiding treatment decisions in patients with pituitary macroadenomas.

## Simple Summary

Pituitary tumors located near the visual pathways can impair vision by affecting the optic nerves, even when they do not produce hormones. In this study, we evaluated whether advanced eye tests can detect early changes in the retina and optic nerve in patients newly diagnosed with these tumors. We followed two groups of patients: one received surgery or medication, while the other was monitored without treatment. Over 12 months, we observed improvements in visual function and changes in specific retinal layers and electrical responses in the treated group. These non-invasive tests may be useful tools for tracking disease progression and assessing the effectiveness of treatment. Our findings may help doctors detect early visual system dysfunction and make better-informed decisions when managing patients with pituitary tumors.

## 1. Introduction

Pituitary adenomas are typically linked to hormone overproduction and/or may also cause symptoms due to the compression of adjacent structures; for example, optic chiasm Pituitary adenomas are typically linked to hormone overproduction and may also cause visual symptoms due to compression of adjacent structures such as the optic chiasm [1,2]. Symptoms depend on the size and location of the adenoma: when its diameter exceeds 10 mm (‘macroadenoma’), it may extend beyond the sella turcica and exert pressure on adjacent structures, potentially leading to neuro-ophthalmologic disorders [3,4,5,6].

Changes in the retinal nerve fiber layer (RNFL) associated with long-standing lesions are also characteristic of pituitary tumors [7,8]. Measuring the circumpapillary retinal nerve fiber layer (RNFL) and the ganglion cell–inner plexiform layer (GCIPL) using optical coherence tomography (OCT) may help predict visual outcomes [9]. Previous studies [10,11] of patients with large pituitary tumors causing optic chiasm compression have identified a diffuse loss of RNFL, even when visual field defects were not present. These studies hypothesize that pituitary tumors themselves, regardless of any compressive effect on the chiasm, may lead to reversible retinal ganglion cell (RGC) dysfunction. It remains unclear whether pituitary tumors not associated with optic chiasm compression can also cause damage to the retinal nerve fiber layer (RNFL).

The photopic negative response (PhNR) is a gradual negative wave of the photopic flash electroretinogram (ERG) that follows the b-wave, offering insights into the function of retinal ganglion cells and their axons. The PhNR may be reduced in conditions that impact the innermost layers of the retina, such as glaucoma and various types of optic neuropathy [12].

Previous studies [13,14,15] on patients with large pituitary tumors causing optic chiasm compression have identified a diffuse loss of RNFL, even in the absence of visual field defects. These studies have also hypothesized that pituitary tumors themselves, even without direct compression of the chiasm, may lead to reversible RGC dysfunction [13,14,15].

The objective of this prospective study was to assess GCIPL and RNFL thicknesses using OCT, along with PhNR via ERG, in a cohort of consecutive de novo patients diagnosed with pituitary macroadenomas. This work offers novel insights by simultaneously evaluating both structural and functional parameters in newly diagnosed patients, providing a comprehensive view of early retinal changes in pituitary macroadenomas. Our findings provide evidence that functional and structural changes in retinal ganglion cells may be detectable even in the absence of visible chiasmal compression, offering new insights into early retinal involvement in pituitary adenoma patients.

## 2. Materials and Methods

This prospective study was conducted at the Department of Endocrinology and the Department of Ophthalmology of a tertiary hospital in Poland. Patients were recruited over 1.5 years, from December 2021 to August 2023, and followed up for 12 months.

The study conformed to the tenets of the Declaration of Helsinki and was approved by the Silesian Ethics Committee, Poland, number PCN/CBN/0022/KB1/124/21.

### 2.1. Study Groups and Treatment Allocation

Participants were recruited from patients admitted to the Endocrinology Department with a newly diagnosed pituitary macroadenoma. None of the patients had received any prior treatment (surgical, pharmacological, or radiotherapeutic) before enrollment in the study.

Patients were classified on the basis of hormonal hypersecretion associated to pituitary tumors: prolactin (PRL)-secreting pituitary tumors; growth hormone (GH)-secreting pituitary tumors; adrenocorticotropic hormone (ACTH)-secreting pituitary tumors, and non-functioning pituitary tumors (NFPAs).

The first group included patients with pituitary macroadenomas requiring treatment and is referred to throughout the study as the treatment group. This group consisted of patients diagnosed with pituitary macroadenoma who underwent either surgical intervention, based on clinical indications such as tumor size and prolactin levels.

The observation group comprised patients with pituitary macroadenoma who did not receive active treatment during the study period and were followed over time. Although not a typical healthy control group, it served as a reference to assess the impact of therapy on the visual system in the treated patients.

At study entry, medical history and duration of symptoms were recorded for each patient. All patients harbored a pituitary macroadenoma at the study entry, confirmed by magnetic resonance imaging (MRI).

During the initial assessment, patients underwent a comprehensive ophthalmic examination, including BCVA, intraocular pressure, refraction, slit-lamp biomicroscopy, dilated stereoscopic fundus examination, posterior segment optical coherence tomography (OCT), and electroretinography. These examinations were repeated after 12 months.

Independent measurements were performed in both groups between the baseline visit and 12 months. Additionally, dependent measurements were performed within groups between the baseline visit and 12 months.

The comparison between groups was conducted to assess the effects of the treatment over time.

The first group received pharmacological and/or surgical treatment for pituitary adenoma, depending on clinical indications. In contrast, the observation group did not receive any treatment during the study period.

### 2.2. Inclusion and Exclusion Criteria

All participants had a best corrected visual acuity (BCVA) of 0.1 or better on the decimal Snellen chart and no underlying ocular or systemic conditions, except for pituitary macroadenoma. Patients were included in the study if they provided informed written consent to participate and had the opportunity to ask questions. Additionally, participants had to be 18 years or older.

Patients were excluded if they had any opacities in the optical media of the eye, including corneal, lens, or vitreous pathologies. Retinal disorders such as retinal degeneration, macular degeneration, diabetic retinopathy, retinal detachment, a history of retinal detachment surgery, traumatic retinal changes, or nystagmus were also exclusion criteria. Individuals with a history of refractive surgery or those who underwent ocular surgery within the last six months were not eligible. Patients with glaucoma, high myopia (defined as spherical equivalent refractive error greater than −6.00 diopters), hyperopia (>+6.00 diopters), psychiatric disorders, or systemic diseases that could prevent the completion of the planned examinations were excluded. Additionally, pregnancy and breastfeeding were considered exclusion criteria.

### 2.3. Optical Coherence Tomography

Optical coherence tomography (OCT) was performed using a Zeiss Cirrus 6000 device (Zeiss, Germany). The Zeiss OCT software version 11.5.2.545332 divides the macular area, including the ganglion cell–inner plexiform layer (GCIPL), into six segments, which are as follows:

1. Superior (S),
2. Nasal Superior (NS),
3. Nasal Inferior (NI)
4. Inferior (I),
5. Temporal Inferior (ST),
6. Temporal Superior (TS).

The Zeiss OCT software divides RNFL into for quadrants:

1. Superior (S),
2. Nasal (N),
3. Inferior (I),
4. Temporal (T).

**Figure 1:**
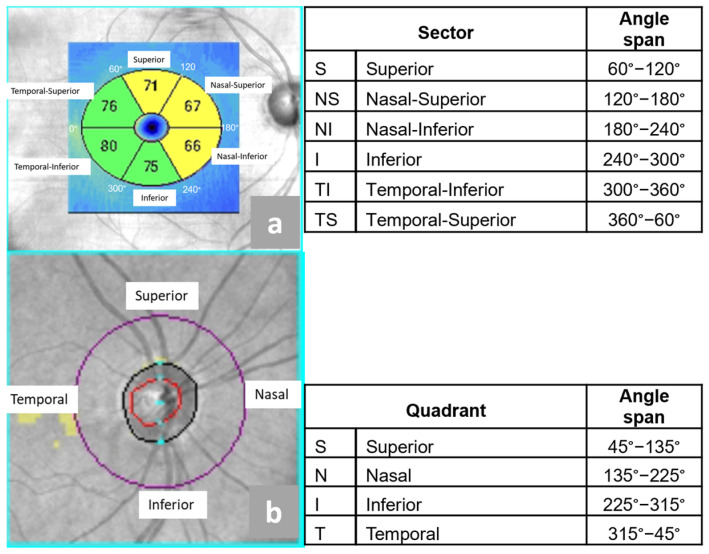
Segmentation of macular GCIPL and peripapillary RNFL quadrants in optical coherence tomography. (**a**) Division of the macular GCIPL into six sectors; (**b**) division of the RNFL around the optic nerve into four quadrants. The figure was created based on clinical data from a representative study participant. The patient provided informed consent for publication. Figure prepared by the first author.

Location and angle spans of the anatomical sectors of macular GCIPL and RNFL around optic disc that were studied, as presented in Figure 1.

### 2.4. Photopic Negative Response in Electroretinography

RETeval (LKC technologies, Gaithersburg, MD, USA) is a portable device used for performing electroretinography (ERG), allowing for a quick and non-invasive evaluation of retinal function. Photopic electroretinography with sensor strips electrodes was performed.

Monocular ERGs were recorded using a Sensor Strip skin electrode (LKC Technologies, Gaithersburg, MD, USA), which incorporates active, reference, and ground electrodes. The strip was placed on the lower eyelid, approximately one millimeter below the lid margin, in accordance with standard guidelines.

**Figure 2:**
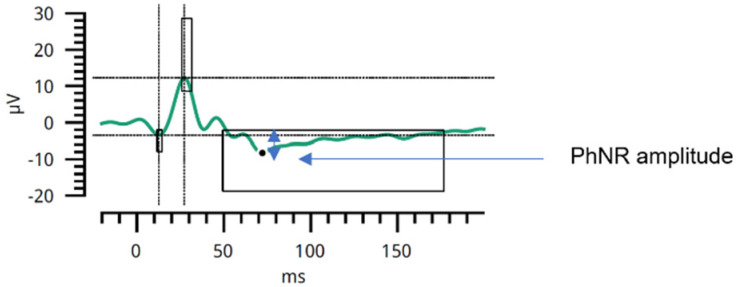
PhNR amplitude. The PhNR amplitude is measured at its minimum, indicated by a black dot. The amplitude is marked with a blue vertical double arrow; μV—microvolts; ms—milliseconds. The figure was created based on clinical data from a representative study participant. The patient provided informed consent for publication. Figure prepared by the first author.

In ERG, parameters such as PhNR amplitude (Figure 2) and W-ratio were analyzed.

The W-ratio was calculated as

(1) $$W\text{-}\mathrm{ratio}=\frac{b-p_{\min}}{b-a}$$

where *b* is the baseline amplitude, *p*_min_ is the minimum amplitude of the negative trough, and *a* is the amplitude of the preceding positive peak. This ratio reflects the relative depth of the PhNR response and helps to standardize measurements across individuals.

### 2.5. Statistical Analysis

The analysis was performed using Statistica 13 (TIBCO Software Inc. (2017). Statistica (data analysis software system), version 13, https://docs.tibco.com/products/spotfire-statistica/archive (accessed on 7 March 2024)).

The distribution of variables in the individual groups was tested using the Shapiro–Wilk test and evaluated graphically by means of histograms. The assumption of homogeneity of variance was checked by the Levene test. The unpaired *t*-test and in justified cases its non-parametric counterpart, the Mann–Whitney U test, was used to compare the results of two independent groups (treatment and observation). In case of repeated measurements (baseline and after 12 months), the paired *t*-test or its non-parametric equivalent, the Wilcoxon signed-rank test, was used. In order to assess the relationship between the variables, the Spearman’s rank correlation coefficients were calculated. A *p*-value less than 0.05 was considered statistically significant.

## 3. Results

### 3.1. Descriptive Statistics

The mean ages of patients in the treatment group and observation groups were 57.30 ± 14.4 and 54.85 ± 17.58 years, respectively.

In the treatment group, the mean age for males (M) was 60.00 ± 14.41 years, while for females (F), the mean age was 53.92 ± 13.62 years.

In the observation group, the mean age for males (M) was 54.50 ± 21.92 years, while for females (F), the mean age was 54.91 ± 17.97 years.

The distribution of pituitary tumor types between the treatment and observation groups is presented in Table 1, where percentages are calculated relative to the rows, columns, and overall sample size.

Table 2 shows the distribution of individuals based on treatment and observation groups categorized by gender (male and female). The data help to evaluate gender proportions in both treatment and observation settings.

Table 3 presents the distribution of individuals undergoing surgical intervention, categorized as “Surgery Yes” or “Surgery No” within the treatment and observation groups. The treatment group is further divided into subcategories “Surgery Yes” (NFPAs-14, PRL-2, ACTH-2, GH-) “Surgery No” with pharmacological treatment using dopamine agonists (PRL-7), to detail the number of cases in each category.

### 3.2. Analytical Statistics

According to the sociodemographic data, an independent *t*-test revealed no significant difference between the groups in terms of age (*p* = 0.6732).

However, there was a notable difference in the male-to-female (M:F) ratio. In the treatment group, the M:F ratio was 15:12, indicating a more balanced distribution, whereas in the observation group, the ratio was 2:11, showing a predominance of females.

#### 3.2.1. Visual Acuity

In the treatment group, the mean BCVA at the initial examination and after 12 months was statistically different (*p* = 0.02). An increase in the mean BCVA value from 0.868 to 0.966 was observed, indicating improvement.

There is no statistically significant difference in visual acuity before and after treatment in the observation group (*p* = 0.524), as presented in Table 4.

#### 3.2.2. Intraocular Pressure

The mean intraocular pressure, measured using Goldmann applanation tonometry, in the treatment group at baseline was 15.48 ± 2.06 mmHg for the right eye and 15.96 ± 3.71 mmHg for the left eye. An independent *t*-test showed no statistically significant difference between the two eyes (t = −0.59, *p* = 0.5682).

The mean intraocular pressure in the observation group at baseline was 16.00 ± 1.53 mmHg for the right eye and 16.08 ± 1.61 mmHg for the left eye. An independent *t*-test showed no statistically significant difference between the two eyes (t = −0.13, *p* = 0.90).

#### 3.2.3. OCT Parameters Versus Parameters in ERG

The comparisons between OCT and ERG parameters in the treatment group are summarized in Table 5.

For the treatment group, statistically significant differences between baseline and after 12 months were observed for RNFL Nasal (*p* = 0.0023), RNFL Inferior (*p* = 0.0004), RNFL Average (*p* = 0.0024), and RNFL Superior (*p* = 0.0073) groups. Additionally, highly significant differences were found for PhNR Amplitude (*p* < 0.0001) and PhNR W-ratio (*p* < 0.0001).

For the treatment group, no statistically significant differences were observed in OCT RNFL in the temporal region (RNFL Temporal), as these fibers are the least exposed to compression associated with pituitary adenoma.

Within the treatment group, the following variables showed a trend toward statistical significance between baseline and after 12 months: GCIPL Average (*p* = 0.0646), GCIPL Temporal Inferior (*p* = 0.0891), and GCIPL Temporal Superior (*p* = 0.0542).

In the observation group, significant changes between baseline and 12 months were observed only for GCIPL Inferior (*p* = 0.0470) and PhNR W-ratio (*p* = 0.0015).

The Mann–Whitney U test revealed statistically significant differences between the treatment and observation groups only for variable RNFL Nasal, both at baseline (*p* = 0.0017) and the after 12 months (*p* = 0.0150).

In contrast, no significant differences were observed between treatment and observation groups in PhNR amplitude and the W-ratio at baseline. However, after 12 months, statistically significant differences emerged for PhNR amplitude (*p* = 0.0012) and the W-ratio (*p* = 0.0016).

**Figure 3:**
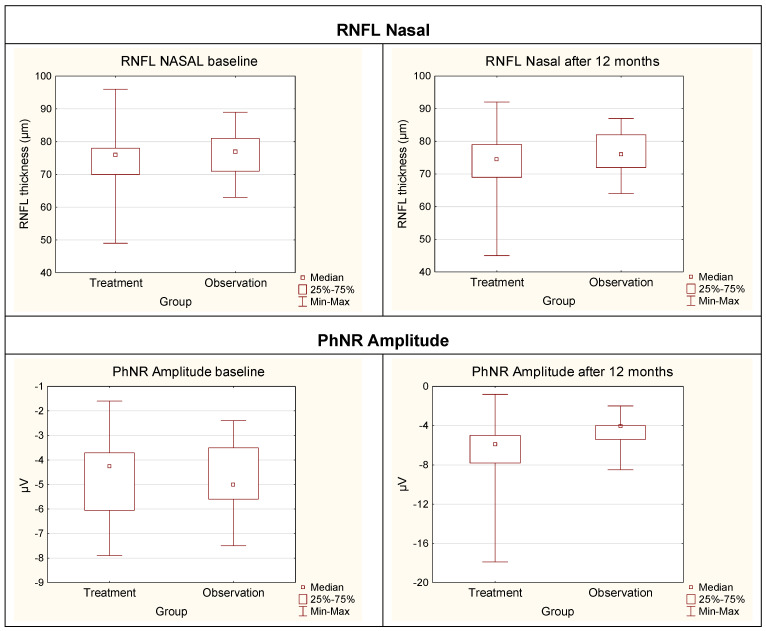
Box-and-whisker plots comparing the treatment group and the observation group for RNFL Nasal and PhNR Amplitude at baseline and after 12 months. RNFL—Retinal Nerve Fiber Layer; PhNR—Photopic Negative Response; μm—micrometers; μV—microvolts; Min–Max—Minium–Maximum.

The box-and-whisker plots (Figure 3) illustrate these findings, demonstrating the distribution of RNFL thickness and PhNR amplitude values across the two groups at both time points.

The variability within the groups, particularly in PhNR amplitude after 12 months, highlights the potential impact of individual differences in disease progression or response to treatment.

These results align with previous studies that have identified RNFL thinning and PhNR amplitude reduction as important indicators of retinal ganglion cell dysfunction in various ocular and neurodegenerative conditions. The observed changes over time suggest that combining structural (RNFL) and functional (PhNR) assessments may provide a more comprehensive understanding of disease progression.

#### 3.2.4. Correlations

At the initial visit in the treatment group, Spearman test correlations were statistically significant between variables such as GCIPL Inferior Temporal and W-ratio. Additionally, GCIPL Superior Temporal, RNFL Average, RNFL Superior, RNFL Nasal, RNFL Inferior, and PhNR Amplitude also showed significant correlations, as presented in Table 6.

At the initial visit in the observation group, Spearman test correlations were statistically significant between variables such as GCIPL Average, GCIPL Superior Nasal, GCIPL Inferior Nasal, GCIPL Inferior, RNFL Inferior, RNFL Temporal, and W-ratio.

Additionally, GCIPL Average, GCIPL Superior Nasal, GCIPL Inferior Nasal, RNFL Temporal, and PhNR Amplitude also showed significant correlations, as shown in Table 6.

In the treatment group, correlations between OCT parameters (GCIPL and RNFL) and ERG parameters (PhNR amplitude and PhNR W-ratio) were few at baseline. However, after 12 months, most of these correlations diminished.

In the observation group, correlations between OCT parameters (GCIPL and RNFL) and ERG parameters (PhNR amplitude and PhNR W-ratio) were numerous at baseline. However, after 12 months, these correlations weakened.

**Figure 4:**
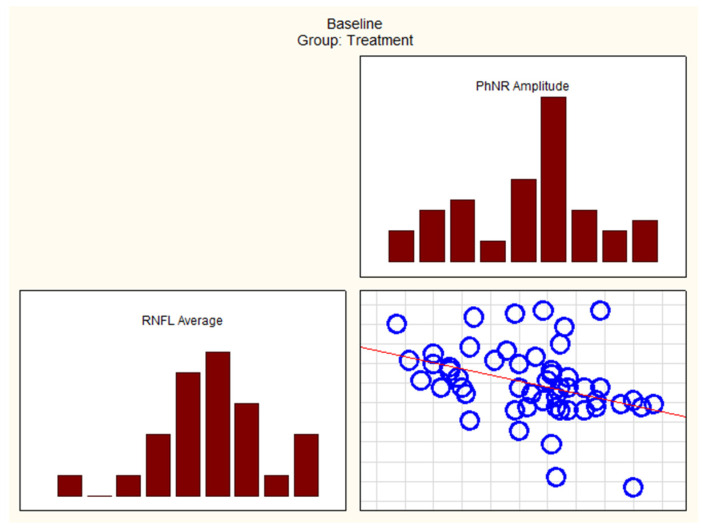
Correlations between variables RNFL Average and PhNR Amplitude at baseline in the treatment group. PhNR—Photopic Negative Response; RNFL—Retinal Nerve Fiber Layer.

**Figure 5:**
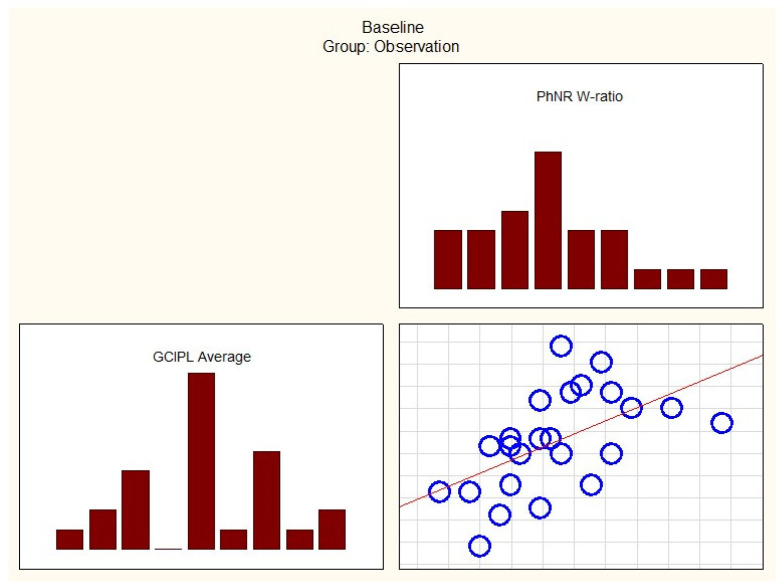
Correlations between variables GCIPL Average and PhNR W-ratio at baseline in the observation group. PhNR—Photopic Negative Response; GCIPL—Ganglion Cell–Inner Plexiform Layer.

Figure 4 and Figure 5 present some of the strongest correlations observed at baseline in the treatment and observation groups, respectively.

## 4. Discussion

Predicting visual outcome following treatment for pituitary macroadenomas is valuable. Patient age, symptom duration, tumor volume, and visual parameters are documented predictors [16].

The extent of retrograde degeneration affecting retinal ganglion cells (RGCs) is considered a key determinant of the final visual outcome [17]. OCT parameters such as GCIPL and RNFL along with PhNR serve as valuable tools for objectively and quantitatively assessing the structural and functional damage to RGCs [18,19,20].

Analysis of GCIPL parameters is particularly difficult in patients with macroadenomas of the pituitary gland because it is unable to determine, based on GCIPL and other parameters, how long the pituitary adenoma may have been present or how long it may have been compressing the optic chiasm [21,22].

This prospective study presents the largest series to date of patients with various histotypes of newly diagnosed pituitary macroadenoma during a one-year follow-up.

According to the literature, the most common types of pituitary macroadenomas are prolactinomas, approximately 25–41% [23] up to 53% [24].

However, in our study, the most common newly diagnosed pituitary macroadenoma admitted for hospitalization at the Department of Endocrinology for detailed assessment was a hormonally non-functioning pituitary adenoma (NFPA). The number of patients with hormonally NFPA was 14 out of 27 (51.8%) in the treated group. In the observation group, all 13 patients (100%) had NFPAs

Several studies have indicated that NFPAs are more prevalent than hormone-secreting ones [25,26]. For example, a nationwide study from Iceland analyzing 410 cases of pituitary adenomas found that 43% were non-functioning, while 40% were prolactin-secreting [27].

NFPAs often remain asymptomatic in the early stages, as they do not cause overt endocrine dysfunction. As a result, they are typically diagnosed at a more advanced stage, when their increasing size leads to mass effects such as visual disturbances or headaches [28]. This delayed presentation may explain why NFPAs were the most commonly detected tumors in our study, as patients were admitted for hospitalization primarily due to symptoms associated with tumor expansion rather than hormonal imbalances.

To our knowledge, there are no studies in the literature on the assessment of PhNR in electrophysiology together with OCT parameters such as GCIPL, RNFL in patients with pituitary macroadenomas. Only one case report presents the use of these parameters in a patient with pituitary macroadenoma during a one-year follow-up [29].

In patients with pituitary tumors, identifying early dysfunction of the visual pathway may prompt adjustments to the medical treatment regimen, potentially reducing the risk of irreversible optic nerve damage [30].

In Table 3, the majority of individuals in the treatment group (74.07%) underwent surgical intervention, with NFPAs (14 cases) being the most common subtype. In contrast, all individuals in the observation group (100%) did not undergo surgery, emphasizing a distinct separation between the two cohorts regarding intervention approaches. This distribution suggests that surgery was predominantly considered for patients in the treatment group, whereas the observation group followed a non-surgical management strategy.

In Table 5, while both groups showed relatively stable values over time, a slight decline in GCIPL and RNFL measurements was observed in the treatment group, whereas the observation group remained more consistent. These findings suggest potential structural changes in the treatment group over time. The PhNR amplitude showed a more pronounced decline in the treatment group (−4.58 to −6.41) over 12 months, whereas it remained relatively stable in the observation group (−4.65 to −4.64). This suggests a potential treatment-related effect on retinal ganglion cell function. Similarly, the W-ratio increased in the treatment group (0.93 to 1.1), while the observation group showed a smaller change (0.91 to 1.0). These differences may indicate adaptive or compensatory mechanisms in response to treatment, which require further exploration.

### 4.1. Treatment Group Versus Observation Group

The findings from the Mann–Whitney U test analysis indicate statistically significant differences in nasal RNFL thickness between the groups at both the initial visit (*p* = 0.0017) and after 12 months (*p* = 0.0150). This suggests that the nasal RNFL parameter may serve as a key biomarker for distinguishing between the groups and monitoring disease progression over time. The persistence of statistical significance across both time points indicates a sustained difference in RNFL thickness, which could be related to the underlying pathology or the effects of treatment in the studied cohort.

Conversely, PhNR amplitude and the W-ratio showed no significant differences between the groups at baseline. However, after 12 months, significant differences emerged for PhNR amplitude (*p* = 0.0012) and W-ratio (*p* = 0.0016). This suggests that changes in retinal ganglion cell function, as reflected by PhNR amplitude, may develop progressively over time rather than being immediately apparent at the initial visit. The delayed emergence of significant differences may indicate that functional impairment lags behind structural changes, reinforcing the importance of longitudinal monitoring in clinical assessments.

Overall, a reduction in GCIPL thickness was observed in the treated group, both in the average value and across segments. However, not all differences were statistically significant. Although no statistically significant differences were observed in the GCIPL Average parameters in the treatment group, a trend toward statistical significance was noted.

The reduction in GCIPL thickness in these patients may have multiple causes.

These changes may be caused by various factors, such as the heterogeneity of tumor size and the different locations of the chiasm. Additionally, since functional changes precede structural changes by about three months, patients could be at different stages of tumor advancement, and the pressure exerted by the tumor on the optic chiasm could vary depending on its duration in each patient.

In our study, among the 27 patients in the treatment group, two had acromegaly, which initially led to an increase in GCIPL parameters above the normal range, followed by a subsequent decrease. While this may explain individual variations in GCIPL changes, the vast majority of patients [31] did not have acromegaly, suggesting that this factor had minimal impact on the overall results.

Although both groups included age-matched patients and no statistically significant differences were observed, the potential influence of age-related variations on GCIPL thickness cannot be entirely excluded.

OCT examination of the posterior segment is a fast, non-invasive, and widely available imaging method for assessing the RNFL and GCIPL. Numerous recent scientific publications confirm its effectiveness. This method is undoubtedly valuable for initial and follow-up assessments in patients with pituitary macroadenomas [32].

However, as our studies confirm, the overall (i.e., average) assessment of RNFL and GCIPL may be insufficient to assess the impact of a macroadenoma on the visual organ before and after treatment in the treatment group. Also, during follow-up, only the analysis of individual squares in the case of RNFL and segments in the case of GCIPL may allow for an accurate assessment of the impact of these tumors on the visual organ, as the nasal fibers of the optic chiasm are primarily the first to be damaged in the course of a pituitary macroadenoma.

Many studies evaluate GCIPL and RNFL before and after surgical treatment of pituitary adenoma but do not specify the type of tumor. In most cases, macroadenoma of the pituitary gland that compress the optic nerves cause a decrease in GCIPL and RNFL values over time [8,14,16,33]. An exception is tumors that produce the growth hormone, which increases the thickness of GCIPL and RNFL [34].

Despite the use of specific inclusion and exclusion criteria, many factors influence the GCIPL and RNFL values. Additionally, there may be inter-individual differences, and the patients are of different ages. As reported by Soh et al., the GCIPL and RNFL gradually decrease with age [35].

The ganglion cell inner plexiform layer (GCIPL) has been reported to show thinning earlier than the retinal nerve fiber layer (RNFL) in cases of chiasmal compression [36,37].

According to Kurian DE et al., the potential for visual acuity recovery may be unrelated to RNFL thickness [16].

Ventura et al. [15] stated that pituitary tumors, even without a compressive effect on the chiasm detected by MRI, can lead to reversible RGC dysfunction. This dysfunction occurs before visual field loss and RGC degeneration, as demonstrated by circumpapillary RNFL in OCT.

RNFL thinning serves as an indicator of axonal degeneration in optic nerve fibers caused by compression; however, it does not directly represent retinal ganglion cell loss [17]. To address this, our study focused on selectively measuring the GCIPL area from macular scans to evaluate both retinal ganglion cell loss and axonal degeneration.

There is a lack of available literature on studies using the RETeval device for assessing PhNR in patients with pituitary macroadenomas. Although classic ERG with DTL electrodes or Jet lenses is widely used in the diagnosis of various ophthalmic disorders, its application in the context of pituitary adenomas has been reported but remains limited [15,30]. An alternative to classic ERG is ERG using skin electrodes with the RETeval device [19,38].

There are no medical literature reports on the use of RETeval for PhNR assessment in these patients.

PhNR is an important parameter in photopic electroretinography for assessing the impact of macroadenomas on the optic nerves. This test is significantly less accessible than OCT and requires a high level of expertise to be conducted and interpreted correctly. Additionally, the cost of the PhNR test includes a disposable electrode, further increasing its overall expense [39].

The staff training process is also longer. Nevertheless, despite its limited availability, this test is extremely valuable in assessing the function of ganglion cells and their axons.

In our study, it is clearly visible—and statistically significant—that in patients in the first group, the PhNR value improved significantly after treatment. Various factors can affect the PhNR result, including patient age, which differs greatly between both groups. Despite many factors influencing the degree of optic nerve impairment, PhNR seems to be a more useful parameter for assessing the effect of macroadenoma treatment on the pituitary gland [15].

This study undoubtedly encourages further research on these patients in a broader group, with additional subdivision based on specific tumor types.

RNFL thickness and GCIPL area measurements using OCT, along with the PhNR W-ratio, may serve as valuable prognostic indicators in the preoperative assessment of chiasmal compression [40]. However, Moon et al. conducted a study considering various tumors compressing the optic chiasm, including not only adenomas but also craniopharyngiomas and meningiomas [40].

In the treatment group, as presented in Table 6, all correlations observed at baseline between OCT parameters (GCIPL, RNFL) and ERG parameters (PhNR amplitude, PhNR W-ratio) completely disappeared after 12 months. This loss of correlation may indicate treatment-induced structural or functional changes, potential neuroplastic adaptations, or variability in individual responses over time. These findings suggest that the initial associations between retinal morphology and electrophysiological function were altered, possibly due to therapeutic intervention.

In the observation group, as shown in Table 6, numerous correlations were initially observed between OCT parameters (GC, PL, and RNFL) and ERG parameters (PhNR amplitude and PhNR W-ratio) at baseline. However, after 12 months, the majority of these associations weakened or disappeared, suggesting potential structural or functional adaptations over time.

Our findings align with previous research indicating that electrophysiological assessments such as the PhNR are valuable in detecting early optic nerve dysfunction. Moon et al. demonstrated that reductions in PhNR amplitude, alongside structural changes observed via OCT, were associated with visual field deficits in patients with chiasmal compression, underscoring the utility of these measures in prognostic evaluations [14].

This supports our findings of increased PhNR amplitude and W-ratio following treatment. On the other hand, regarding structural integrity, Iqbal et al. and Lee et al. reported that RNFL thinning often persists or progresses despite surgical intervention, reflecting irreversible damage caused by chronic compression [21,41]. These observations are consistent with our results, where we observed ongoing RNFL thinning even though functional parameters improved. Together, these comparisons reinforce the complementary role of structural and functional assessment in managing patients with pituitary macroadenomas.

The presence of pituitary macroadenoma, even without obvious compression or modelling of the optic chiasm on magnetic resonance imaging, may still negatively impact the function and structure of nerve cells, as evidenced by changes in OCT and ERG parameters. The disappearance of these correlations may support decision-making regarding surgical treatment for patients in the observational group.

These findings confirm that the assessment of structural changes in OCT should be performed alongside functional evaluation using PhNR both amplitude and W-ratio in the assessment of patients with pituitary macroadenomas.

### 4.2. Discussion Summary

The conducted study shows that patients in the treatment group, whether receiving pharmacological or surgical intervention, generally exhibited a trend of worsening GCIPL and RNFL parameters while demonstrating an improvement in PhNR W-ratio ratio parameters, even though not all differences reached statistical significance

In the observation group, patients did not undergo any treatment and were solely monitored over time. While OCT and ERG parameters remained statistically stable, correlations between these parameters were present at baseline but disappeared in many cases after 12 months of observation. This indicates that changes in these parameters occurred. This may be related to the pituitary adenoma, which can still affect the optic chiasm despite the stabilization observed in magnetic resonance imaging.

Proper radiological, endocrinological, and ophthalmological evaluation is essential for assessing patients with macroadenomas. Multidisciplinary collaboration is particularly important in determining the need for surgical treatment, especially for hormonally inactive adenomas. This approach ensures accurate qualification and optimal patient management.

### 4.3. Limitations and Strengths of the Study

The main strength of this study is its prospective design. Another strength is that most of our subjects are local patients, ensuring a consistent follow-up period. The treatment group has a more balanced distribution of males and females, while the observation group is female-dominated. In the observation group, the male-to-female ratio is 2:11, which indicates that the groups are not equal in size. There are significantly more females than males, meaning the gender distribution is imbalanced.

### 4.4. Clinical Implications

- Treatment group:

In the treatment group, the effects of therapy can be positive (improved structure and function of the optic nerve) or negative (irreversible damage or complications). An individualized approach to patients is essential, particularly in selecting the appropriate treatment method, considering the patient’s age, and ensuring early detection of changes in OCT and ERG.

In pharmacological treatment (e.g., somatostatin analogues, dopamine agonists), side effects may occur that could indirectly impact visual function, such as changes in intraocular pressure or alterations in the blood supply to the optic nerve.

In surgical treatment, the impact on visual function can vary, with potential improvement due to relief of optic chiasm compression, but also a risk of complications, such as optic nerve damage or vascular disturbances, which may affect long-term visual outcomes.

- Observation group:

The study results suggest that patients with hormonally inactive pituitary macroadenomas may exhibit progressive changes in the GCIPL and RNFL, even without treatment. Therefore, regular ophthalmological monitoring may be necessary to track potential damage to neural structures. Additionally, individualized therapy, considering the patient’s age and the dynamics of changes in ERG, may improve treatment effectiveness.

Further research is needed to explore the clinical implications of these findings, particularly in relation to disease staging, treatment efficacy, and potential prognostic value. Longitudinal studies with larger sample sizes could help validate these results and refine the role of RNFL thickness and PhNR amplitude as diagnostic and monitoring tools.

## 5. Conclusions

In patients with pituitary macroadenoma, active treatment led to a notable improvement in BCVA, alongside increases in PhNR amplitude and W-ratio, despite concurrent reductions in RNFL thickness across multiple quadrants. In contrast, patients managed with observation alone exhibited only minimal changes, primarily limited to minor alterations in GCIPL thickness and W-ratio. Comparative analysis underscored the benefits of active treatment over observation, demonstrating better preservation of optic nerve function, even though progressive retinal structural thinning was observed in treated patients. These findings quantitatively and qualitatively highlight the effectiveness of treatment for pituitary macroadenomas in enhancing retinal ganglion cell function while acknowledging ongoing structural deterioration over time.

Moreover, combining structural (OCT) and functional (ERG PhNR) parameters may provide valuable prognostic information and support early treatment decisions, particularly when structural abnormalities are subtle or not yet widespread.

## Figures and Tables

**Table 1:** Percentages are calculated relative to the rows, columns, and overall sample size.

Group	NFPAs	Prolactinoma (PRL)	ACTH-Secreting Tumor	GH-Secreting Tumor	Row Total
Treatment	14	9	2	2	27
% Row	51.85%	33.33%	7.41%	7.41%	-
% of Total	35.00%	22.50%	5.00%	5.00%	67.50%
Observation	13	0	0	0	13
% Row	100.00%	0.00%	0.00%	0.00%	-
% of Total	32.50%	0.00%	0.00%	0.00%	32.50%
Total	27	9	2	2	40
% of Total	67.50%	22.50%	5.00%	5.00%	100.00%

NFPAs—non-functioning pituitary adenomas; PRL—prolactinoma; ACTH—adrenocorticotropic hormone-secreting tumor; GH—growth hormone-secreting tumor.

**Table 2:** Distribution of treatment and observation by gender.

Group	Male	Female
Treatment	15	12
%	55.56%	44.44%
Observation	2	11
% of Row	15.38%	84.62%
Total	17	23
% of Total	42.50%	57.50%

Distribution of individuals by gender (male and female) in treatment and observation groups. The table includes the count of individuals, percentages within rows, and total percentages to provide a comprehensive view of gender representation within each group.

**Table 3:** Distribution of treatment and observation by surgical intervention (yes/no) in the treatment group and observation group.

Group	Surgery Yes	Surgery No	Row Total
Treatment	20 Within: NFPAs-14 PRL-2 ACTH-2 GH-2	PRL-7 (dopamine agonists: cabergoline bromocriptine)	27
% of Row	74.07%	25.93%	
Observation	0	13	13
% of Row	0.00%	100.00%	

Distribution of individuals undergoing surgical intervention (“Surgery Yes” or “Surgery No”) within the treatment and observation groups. The treatment group is further divided into subcategories (NFPAs, PRL, ACTH, GH). Percentages of rows (% of Row) illustrate the proportional distribution across surgical outcomes, while row totals provide a comprehensive overview of the data distribution. NFPAs—non-functioning pituitary adenomas; PRL—prolactinoma; ACTH—adrenocorticotropic hormone-secreting tumor; GH—growth hormone-secreting tumor.

**Table 4:** Change in visual acuity—Wilcoxon signed-rank test (due to non-normal distribution).

Distant Snellen BCVA	At Baseline Mean (SD)	After 12 Months Mean (SD)	*p*-Value
Treatment group	0.868 (0.270)	0.966 (0.137)	0.0233
Observation group	0.904 (0.216)	0.915 (0.201)	0.524

SD—standard deviation; BCVA—best corrected visual acuity.

**Table 5:** Longitudinal Analysis of OCT and ERG Parameters in the Treatment Group and the Observation Group.

Variable	Group (Treatment/ Observation)	Baseline (0 Months) Median	Baseline (0 Months) IQR (Q1–Q3)	After 12 Months Median	After 12 Months IQR (Q1–Q3)	*p*-Value (Between 0 & 12 Months)—Important Values
GCIPL Average	Treatment	76	(70–78)	74.5	(69–79)	*p* ** = 0.0646
	Observation	77	(71–81)	76	(72–82)	
GCIPL Superior	Treatment	75	(70–81)	73	(67–81)	
	Observation	76.5	(74.5–83)	76	(74–83)	
GCIPL Nasal Superior	Treatment	75	(66–80)	73.5	(65–79)	
	Observation	78	(68–83)	77	(67–83)	
GCIPL Nasal Inferior	Treatment	75	(65–79)	72	(65–80)	
	Observation	77	()	77	()	
GCIPL Inferior	Treatment	75	(68–77)	73	(68–76)	
	Observation	76.5	(72–80)	75.5	(71–79.5)	*p* * = 0.0470
GCIPL Temporal Inferior	Treatment	78	(74–81)	78	(72–80)	*p* ** = 0.0891
	Observation	79	(72–84)	79	(72–84)	
GCIPL Temporal Superior	Treatment	77	(72–81)	76	(72–80)	*p* ** = 0.0542
	Observation	79	(75–81)	78	(74–82)	
RNFL Average	Treatment	87	(81–95)	84	(81–91)	*p* * = 0.0024
	Observation	83	(79–93)	84	(78–91)	
RNFL Superior	Treatment	110	(100–119)	106.5	(96–118)	*p* * = 0.0073
	Observation	105	(97–112)	104.5	(99.5–112.5)	
RNFL Nasal	Treatment	72	(66–79)	68	(62–78)	*p* * = 0.0023
	Observation	64	(58–71)	64	(58.5–70)	
RNFL Inferior	Treatment	114.5	(99–126)	109.5	(100–121)	*p* * = 0.0004
	Observation	114	(100–126)	115	(104–125)	
RNFL Temporal	Treatment	58	(50–64)	57	(47–62)	
	Observation	55	(50–69)	58	(51–72)	
PhNR Amplitude	Treatment	−4.3	(−6.1 to −3.7)	−5.9	(−7.8 to −5)	*p* * < 0.0001
	Observation	−5/.0	(−5.6 to −3.5)	−4.1	(−5.4 to 4)	
W-ratio	Treatment	0.91	(0.86–0.98)	1.10	(1.03–0.15)	*p* * < 0.0001
	Observation	0.90	(0.87–0.95)	1.00	(0.95–1.04)	*p* = 0.0015

*p* *—statistically significant result with *p* < 0.05; *p* **—there is a tendency toward statistical significance.

**Table 6:** All significant correlations at baseline in the treatment group and the observational group.

Variable Pair	R Spearman	*p*-Value
Treatment Group		
GCIPL Inferior Temporal and PhNR W-ratio	0.3095	0.0287
GCIPL Superior Temporal and PhNR Amplitude	−0.2865	0.0415
RNFL Average and PhNR Amplitude	−0.4054	0.0032
RNFL Superior and PhNR Amplitude	−0.3133	0.0237
RNFL Nasal and PhNR Amplitude	−0.3655	0.0077
RNFL Inferior and PhNR Amplitude	−0.3682	0.0072
Observational Group		
GCIPL Average and PhNR Amplitude	−0.39690	0.04947
GCIPL Average and PhNR W-ratio	0.63291	0.00068
GCIPL Superior Nasal and PhNR Amplitude	−0.45932	0.02089
GCIPL Superior Nasal and PhNR W-ratio	0.71630	0.00005
GCIPL Inferior Nasal and PhNR Amplitude	−0.54568	0.00478
GCIPL Inferior Nasal and PhNR W-ratio	0.72132	0.00047
GCIPL Inferior and PhNR W-ratio	0.63787	0.00079
RNFL Inferior and PhNR W-ratio	0.59098	0.00186
RNFL Temporal and PhNR Amplitude	−0.45144	0.02349
RNFL Temporal and PhNR W-ratio	0.54597	0.00475

This table presents all statistically significant correlations (*p* < 0.05) at baseline in the treatment group and the observational group. GCIPL—Ganglion Cell–Inner Plexiform Layer; RNFL—Retinal Nerve Fiber Layer; PhNR—Photopic Negative Response.

## Data Availability

The original contributions presented in this study are included in the article. Further inquiries can be directed to the corresponding author.

## Abbreviations

The following abbreviations are used in this manuscript:

GCIPL	Ganglion Cell–Inner Plexiform Layer
RNFL	Retinal Nerve Fiber Layer
PhNR	Photopic Negative Response
PhNR W-ratio	Photopic Negative Response W-Ratio
OCT	Optical Coherence Tomography
GH	Growth Hormone
ACTH	Adrenocorticotropic Hormone
NFPAs	Non-Functioning Pituitary Tumors
ERG	Electroretinography
RGC	Retinal Ganglion Cell
BCVA	Best Corrected Visual Acuity
S	Superior
TS	Temporal Superior
TI	Temporal Inferior
NS	Nasal Superior
NI	Nasal Inferior
I	Inferior
T	Temporal
N	Nasal
MRI	Magnetic Resonance Imaging
ms	Milliseconds
μV	Microvolts
Min–Max	Minium–Maximum
